# AB-Kefir Reduced Body Weight and Ameliorated Inflammation in Adipose Tissue of Obese Mice Fed a High-Fat Diet, but Not a High-Sucrose Diet

**DOI:** 10.3390/nu13072182

**Published:** 2021-06-24

**Authors:** Yung-Tsung Chen, Ai-Hua Hsu, Shiou-Yun Chiou, Yu-Chun Lin, Jin-Seng Lin

**Affiliations:** 1 Culture Collection & Research Institute, SYNBIO TECH INC., Kaohsiung City 821, Taiwan; ianchen619@gmail.com (Y.-T.C.); ethsu1234@synbiotech.com.tw (A.-H.H.); shiouyun@synbiotech.com.tw (S.-Y.C.); 2 Livestock Research Institute, Council of Agriculture, Executive Yuan, Tainan City 712, Taiwan; hiujj@mail.tlri.gov.tw

**Keywords:** obesity, inflammation, gut microbiota, probiotics

## Abstract

Consumption of different types of high-calorie foods leads to the development of various metabolic disorders. However, the effects of multi-strain probiotics on different types of diet-induced obesity and intestinal dysbiosis remain unclear. In this study, mice were fed a control diet, high-fat diet (HFD; 60% kcal fat and 20% kcal carbohydrate), or western diet (WD; 40% kcal fat and 43% kcal carbohydrate) and administered with multi-strain AB-Kefir containing six strains of lactic acid bacteria and a *Bifidobacterium* strain, at 10^9^ CFU per mouse for 10 weeks. Results demonstrated that AB-Kefir reduced body weight gain, glucose intolerance, and hepatic steatosis with a minor influence on gut microbiota composition in HFD-fed mice, but not in WD-fed mice. In addition, AB-Kefir significantly reduced the weight and size of adipose tissues by regulating the expression of *CD36*, *Igf1*, and *Pgc1* in HFD-fed mice. Although AB-Kefir did not reduce the volume of white adipose tissue, it markedly regulated *CD36*, *Dgat1* and *Mogat1* mRNA expression. Moreover, the abundance of *Eubacterium_coprostanoligenes_*group and *Ruminiclostridium* significantly correlated with changes in body weight, liver weight, and fasting glucose in test mice. Overall, this study provides important evidence to understand the interactions between probiotics, gut microbiota, and diet in obesity treatment.

## 1. Introduction

Fermented foods have a long history of use and have shown beneficial effects in humans. Among fermented foods, kefir has been distinguished as an important food conferring various health benefits, including anti-pathogens [1], anti-inflammation [2], and anti-tumor properties [3], as well as improving lactose tolerance [4], lowering cholesterol [5], and ameliorating fatty liver disease [6] and obesity [7]. Therefore, these characteristics of kefir have attracted researchers to evaluate its functional properties that have not been understood till date.

Traditional homemade kefir is composed of various bacterial species; however, different culture conditions often lead to inconsistent effects and contamination. Therefore, rigorous production of kefir probiotic strain is required to control the quality, and it was demonstrated that manufactured and traditional kefir showed similar efficacies [8]. *Lactobacillus* and *Bifidobacterium* strains are most commonly used in fermented foods and probiotic supplements. In our previous studies, six lactic acid bacteria and one *Bifidobacterium* strain at certain concentrations in kefir (AB-Kefir) showed no detrimental effects. Kefir also aided in improving exercise performance by modulating the gut microbiota composition in mice [9] and ameliorated gastrointestinal symptoms, such as abdominal pain and bloating, in adults [10]. However, the effects of these strains on obesity remain unknown.

Lifestyle changes and excessive intake of high-calorie diet contributes to the pathogenesis of obesity and metabolic disorders. Previous studies demonstrated that a high-fat diet caused hypertension [11] and hyperleptinemia [12] and a high-sucrose diet caused insulin resistance [13] and glucose intolerance [14] in a rodent model. Such diets regulate the expression of genes involved in lipid metabolism, resulting in inflammation of the liver and white adipose tissue (WAT) in mice [15]. The gut microbiome is considered as a critical organ that plays an important role in obesity [16]; on being manipulated by a diet, this organ alters the intestinal microbiome and metabolome in obese subjects [17,18]. Therefore, the association between diet and gut microbiota has been indicated as a necessary factor in obesity treatment and its complications.

In the present study, we examined the effect of AB-Kefir on high-fat or high-sucrose diet-induced obesity in mice. We further investigated changes in the expression of lipid metabolism-related genes and gut microbiota regulated by AB-Kefir intervention in different diets.

## 2. Materials and Methods

### 2.1. Preparation of Probiotic Strains

AB-Kefir was provided from SYNBIO TECH INC (Kaohsiung, Taiwan). The reference AB-Kefir in this study was a product containing six lactic acid bacteria and one *Bifidobacterium* in a lyophilized form, with a total cell count of 10^11^ CFU/g. The new official names of the strains are [19]: *B. longum* BL986, *Lactobacillus* (*L.*) *acidophilus* LA1063, *L. fermentum* LF26 (*Limosilactobacillus fermentum*), *L. helveticus* LH43, *L. paracasei* ssp. *paracasei* LPC12 (*Lacticaseibacillus paracasei* ssp. *paracasei*), *L. rhamnosus* LRH10 (*Lacticaseibacillus rhamnosus*), and *Streptococcus thermophilus* ST30.

### 2.2. Experimental Design of the Animal Model

Seven-week-old male C57BL/6 mice were purchased from BioLASCO Taiwan Co., Ltd. (Taiwan). The mice were maintained under a 12 h light–dark cycle and provided food and water *ad libitum*. Mice were randomly grouped into five groups. The control diet (CD), high-fat diet (HFD), and western diet (WD) groups were fed a calorie-from-fat diet of 10% (D12450B, Research Diets Inc., New Brunswick, NJ, USA), 60% (D12492, Research Diets), and 40% (D12079B, Research Diets), respectively, and all the groups were administered saline by gavage daily for 10 weeks. The other two groups were administered AB-Kefir (ABK) at 10^9^ CFU/mouse/day, with the mice being fed either a HFD (HFABK) or WD (WDABK) for 10 weeks. The components of the diets are listed in Table S1. Body weight gain and calorie intake were recorded weekly during the experiment. After 10 weeks of feeding, all the mice were made to fast overnight, anesthetized with isoflurane, and sacrificed for sample collection. Liver and adipose tissues, including epididymal WAT (eWAT), inguinal WAT (iWAT), and retroperitoneal WAT (rpWAT), from mice were weighed and stored at −80 °C for future analysis. All animal husbandry obeyed the national legal requirements, and experimental protocols were approved by the Institutional Animal Care and Use Committee of Livestock Research Institute (permit no. LRI IACUC108-42). Serum glucose and triglyceride levels were measured at the Accuspeed Medical Laboratory (Tainan, Taiwan).

### 2.3. Oral Glucose Tolerance Test (OGTT)

To determine glucose tolerance in mice, an OGTT was performed as described by Andrikopoulos et al. [20], with some modifications. Briefly, after feeding the mice with the above-mentioned diets for 8 weeks, mice were fasted overnight and treated with 2 g of glucose/kg body weight by gavage. Blood samples were harvested from the tails of mice at 0, 15-, 30-, 60-, and 120-min post-glucose treatment, and glucose levels were measured using a blood glucose meter (Accu-Chek^®^ Performa, Roche Diabetes Care Inc., Rotkreuz, ZUG, Switzerland) following the manufacturer’s instructions. The area under the curve (AUC) of glucose level was calculated using GraphPad Prism 8.0 (GraphPad Software Inc., San Diego, CA, USA). 

### 2.4. Fecal Short Chain Fatty Acid (SCFA) Levels

The levels of fecal SCFAs in mice were analyzed as described by Torii et al. [21], with some modifications. Briefly, after feeding the mice with appropriate diets for 10 weeks, their fecal samples were collected. SCFAs in feces were extracted using 70% ethanol solution. The extracted solution was mixed with 2-ethylbutyric acid (109959, Sigma-Aldrich Co., St. Louis, MO, USA) as an internal standard. Next, a reaction-assistive agent was added, and the mixture was reacted at 60 °C for 20 min. The reaction was terminated with potassium hydroxide (30,603, Sigma-Aldrich) solution; the reaction mixture was incubated at 60 °C for 20 min and then extracted with a phosphoric acid solution (B0992, Katayama Chemical Industries Co., Ltd., Osaka, Japan) and ether. The upper ether layer was collected and mixed with water for further extraction. Finally, an ether layer was obtained and air-dried. Methanol was added to dissolve fatty acid hydrazide present in the sample. The final product was analyzed using high-performance liquid chromatography (HPLC). HPLC was performed using a LaChrom L-7100 HPLC system (Hitachi Ltd., Tokyo, Japan) equipped with a NUCLEODUR C18 HTec column (MACHEREY-NAGEL, Düren, Germany) and a UV-VIS detector (L-7420, Hitachi).

### 2.5. Histological Staining

Adipose tissues (eWAT, iWAT, and rpWAT) and liver tissues from mice were harvested and fixed with 4% formalin, followed by a standard histological protocol, and then stained with hematoxylin and eosin (H&E). Histological staining was carried out by the Scientific Integration Design Service Corporation (Kaohsiung, Taiwan). Cross-sectional areas of the adipocytes were calculated using ImageJ software. The hepatocellular ballooning score was determined for discriminating the levels of ballooning degeneration (0: none, 1: few balloon cells; 2: many cells or prominent ballooning) using a diagnostic criterion of nonalcoholic fatty liver disease [22]. The hepatic triglyceride content was determined using a triglyceride colorimetric assay kit (10,010,303, Cayman Chemical Company Inc., Ann Arbor, MI, USA), following the manufacturer’s instructions.

### 2.6. Real-Time Polymerase Chain Reaction (qPCR) for mRNA Quantitation

To determine the expression of lipid metabolism-related genes in eWAT, total RNA was purified from the samples using the QIAzol lysis reagent (79,306, QIAGEN, Hilden, Germany) in accordance with the manufacturer’s protocol. cDNA was made from 1 μg of total RNA using a High-Capacity cDNA Reverse Transcription kit with RNase inhibitor (437,4966, Applied Biosystems, Thermo Fisher Scientific Co., Waltham, MA, USA). The target gene levels were determined using qPCR with PowerUp™ SYBR™ Green Master Mix (A25741, Applied Biosystems). The results were analyzed using QuantStudio^TM^ 3 real-time PCR systems (Applied Biosystems) for qPCR analyses. Relative quantification of target gene expression was calculated by using the 2^-ΔΔCT^ method and normalized to an housekeeping gene, glyceraldehyde-3-phosphate dehydrogenase (*GAPDH*). RT-qPCR results were performed as fold differences relative to a relevant control sample. The primer sequences are listed in Table S2.

### 2.7. 16S rRNA Sequencing

The genomic DNA of bacteria in feces from test mice was isolated using a QIAamp DNA Stool kit (QIAGEN) in accordance with the manufacturer’s instructions. The variable region V3–V4 of 16S ribosomal RNA was amplified by PCR using primers with a sample-specific barcode (F:5′-TCGTCGGCAGCGTCAGATGTGTATAAGAGACAGCCTACGGGN GGCWGCAG-3′ and R:5′-GTCTCGTGGGCTCGGAGATGTGTATAAGAGACA GGACTACHVGGGTATCTAATCC-3′) for microbiome evaluation, as described previously [23]. Sequencing was performed by using an Illumina MiSeq sequencing platform, following the manufacturer’s instructions. The noisy raw reads were filtered using QIIME (v1.9.1) [24] to obtain effective tags. Operational taxonomic units (OTUs) were clustered from representative sequences using UPARSE software (v7.0.1090) [25]. The OTUs were assigned at a ≥97% sequence similarity threshold. Taxonomic information was based on the RDP classifier (v2.2) [26] with a confidence cutoff of 0.8. 16S rRNA analysis was performed using PyNAST (v1.2) [27], Greengenes (gg_13_8, default) [28], Silva (v132) [29], and NCBI databases. Chao1 and ACE indices of alpha diversity and principal component analysis (PCA) plots were performed using QIIME (v1.9.1) and R software (v3.3.1). Taxonomic cladograms were illustrated using the linear discriminant analysis effect size (LEfSe) method [30] with linear discriminant analysis (LDA) > 4 and significance at *p* < 0.05 using Kruskal–Wallis test. Spearman’s correlations of six parameters (body weight, body weight gain, serum triglycerides, total weight of WAT, weight of liver, and fasting glucose) and top 10 most abundant intestinal genera were calculated using GraphPad Prism software.

### 2.8. Statistical Analyses

All statistical analyses were calculated using GraphPad Prism 8.0. The data are expressed as mean ± standard deviation (SD). Significant differences among the experimental results were estimated using one-way ANOVA with post hoc Tukey’s test or non-parametric Kruskal–Wallis test with post hoc Dunn’s multiple comparison test. Statistical significance was set at *p* < 0.05.

## 3. Results

### 3.1. AB-Kefir Ameliorated Body Weight Gain in HFD-Fed, but Not in WD-Fed, Mice

**Figure 1:**
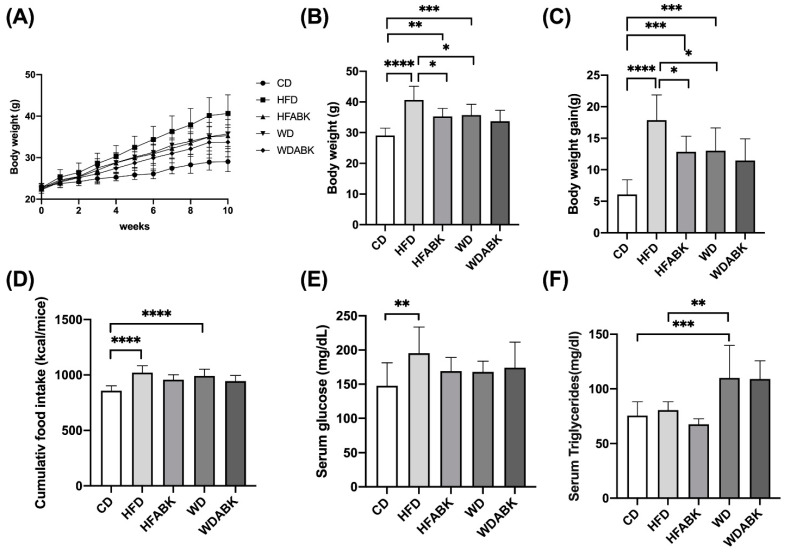
Effect of AB-Kefir on obesity in HFD- and WD-fed mice. Effect of AB-Kefir on (**A**) body weight for 10 weeks, (**B**) body weight at week 10, (**C**) body weight gain, (**D**) cumulative food intake, and serum levels of fasting (**E**) glucose and (**F**) triglycerides. Data are expressed as mean ± S.D. (n = 10 per group) * *p* < 0.05, ** *p* < 0.01, *** *p* < 0.001, **** *p* < 0.0001. Statistics were analyzed by using one-way ANOVA with Tukey’s post hoc test.

Probiotic strains were administered at 10^9^ CFU/mouse/day to HFD- or WD-fed mice for 10 weeks, to evaluate their anti-obesity effects. As shown in Figure 1A–C, HFD and WD groups showed a significant increase in body weight and body weight gain with higher accumulative calorie intake compared to the CD group. Significantly increased serum glucose levels were seen in HFD-fed mice, whereas WD-fed mice showed no change (Figure 1E). However, serum triglyceride levels in the WD-fed group were higher than those in CD and HFD groups (Figure 1F). In the AB-Kefir-administered groups, probiotics significantly decreased body weight and body weight gain in HFD-fed mice, but not in WD-fed mice. However, the AB-Kefir-administered group did not show significant changes in serum glucose and triglyceride levels, and accumulative calorie intake compared with HFD- and WD-fed groups. Therefore, we speculated that the effect of AB-Kefir on the reduction of body weight in mice was due to the impact on energy balance since energy expenditure and fat absorption were not measured.

### 3.2. AB-Kefir Improved Glucose Intolerance in HFD-Fed, but Not in WD-Fed, Mice

**Figure 2:**
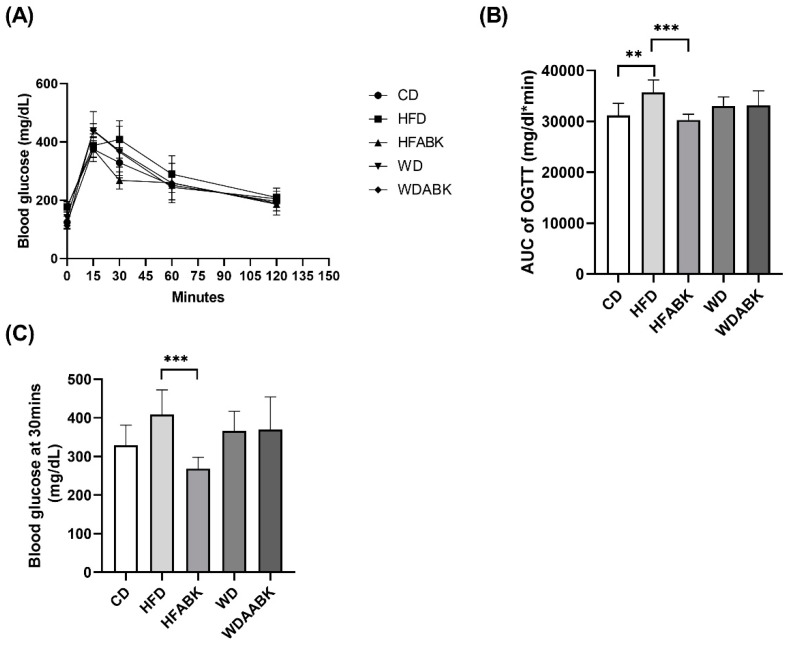
Effect of AB-Kefir on glucose tolerance test in HFD- and WD-fed mice. Overnight-fasted mice were administered 2.0 g/kg BW glucose by gavage. Blood samples were taken at 0, 15-, 30-, 60-, and 120-min post-administration to measure the levels of blood glucose. (**A**) Curve of OGTT, (**B**) AUC of OGTT, and (**C**) glucose level at 30 min of OGTT are shown. Data are expressed as mean ± SD (n = 8 per group). ** *p* < 0.01, *** *p* < 0.001. Statistics were analyzed by using one-way ANOVA with Tukey’s post hoc test.

To evaluate the effect of probiotics on glucose tolerance in different diet-induced obese mice, an OGTT was performed. Compared with the CD group, the HFD group showed a significant increase in the AUC of glucose level in the OGTT; however, the WD group showed no difference in AUC (Figure 2A,B). In contrast, AB-Kefir administration significantly reduced the AUC of glucose levels in HFD-fed mice, but not in WD-fed mice. Moreover, glucose levels in mice fed with HFD were slightly higher than in those fed with CD and WD (Figure 2C). AB-Kefir administration significantly reduced the glucose levels at 30 min after administration of glucose in mice fed with HFD, but not in the WD-fed group. Thus, results of the OGTT demonstrated that administration of probiotics specifically improved glucose intolerance in HFD-induced obese mice. 

### 3.3. AB-Kefir Ameliorated Hepatic Steatosis in HFD-Fed, but Not in WD-Fed, Mice 

**Figure 3:**
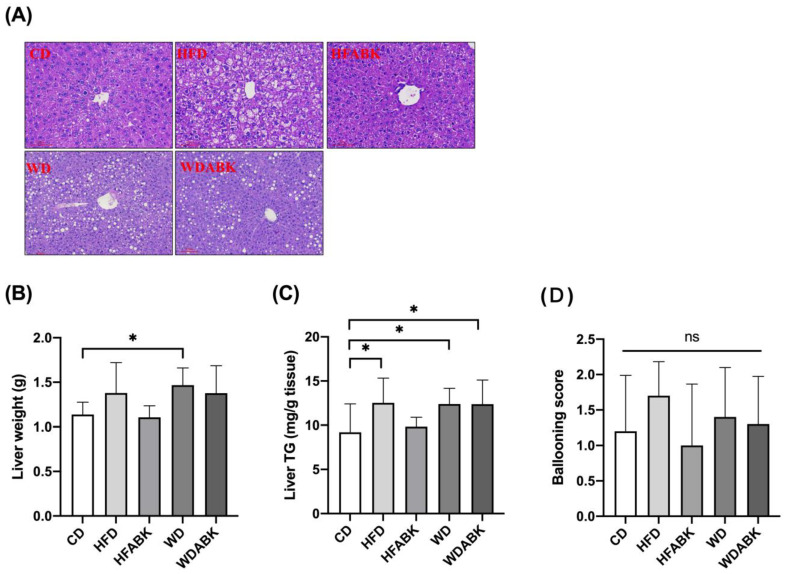
Effect of AB-Kefir on hepatic steatosis in HFD- and WD-fed mice. (**A**) H&E staining of representative histological sections of the liver tissue. Scale bar = 100 µm. (**B**) Liver weights and (**C**) hepatic triglyceride levels and (**D**) hepatocellular ballooning score are shown. Data are expressed as mean ± SD (n = 10 per group). * *p* < 0.05. ns: not significant. Statistics were analyzed by using one-way ANOVA with Tukey’s post hoc test.

Obesity is accompanied by an increased risk of NAFLD [31]. To examine the ability of probiotics to improve hepatic steatosis in HFD- and WD-fed mice, histological sections of the liver tissues were stained with H&E. The hepatocellular ballooning score was determined. Hepatic triglyceride content was also determined. As shown in Figure 3, HFD feeding induced a higher hepatocellular ballooning score and increased hepatic triglyceride levels compared to the CD group. The hepatocellular ballooning score and triglyceride content were observed to be more intense in the liver tissues of the WD group than in those of the CD group. Moreover, administration of AB-Kefir slightly reduced the triglyceride content and hepatocellular ballooning score in the liver tissues of HFD-fed and WD-fed mice, but there was no significant difference. 

### 3.4. AB-Kefir Attenuated Adipocyte Hypertrophy and Regulated the Expression of Genes Related to Lipid Metabolism and Inflammation in the WAT of HFD-Fed Mice 

**Figure 4:**
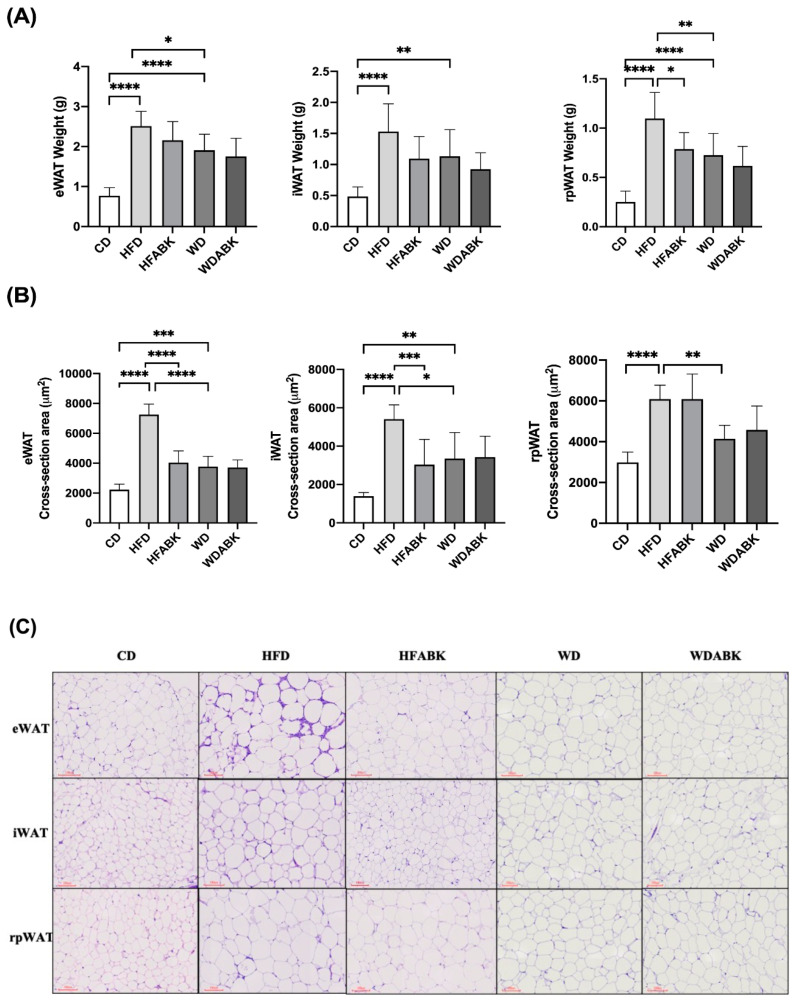
Effect of AB-Kefir on adipogenesis of white adipose tissue (WAT) in HFD- and WD-fed mice. (**A**) Weight and (**B**) cross-sections of epididymal, inguinal, and retroperitoneal WAT are shown. (**C**) H&E staining of representative histological sections of the WAT tissues. Scale bar = 100 µm. Data are expressed as mean ± S.D. (n = 8 per group) * *p* < 0.05, ** *p* < 0.01, *** *p* < 0.001, **** *p* <0.0001. Statistics were analyzed by using one-way ANOVA with Tukey’s post hoc test.

Because adipocyte hypertrophy is a characteristic of obesity, the tissue weight and volume of adipocytes in WAT of test mice were quantified. As shown in Figure 4A,B, compared to those in the CD group, tissue weight and cross-sectional area of adipocytes in eWAT, rpWAT, and iWAT were significantly increased by both HFD and WD. Moreover, the tissue weight and cross-sectional area of adipocytes in eWAT and rpWAT in HFD were higher than those in the WD group. In addition, a higher cross-sectional area of iWAT adipocytes was observed in the WD group. In contrast, AB-Kefir administration not only significantly reduced the tissue weight of iWAT but also decreased the cross-sectional area of adipocytes in eWAT and rpWAT in HFD-fed mice; however, AB-Kefir did not show a significant difference in these parameters in WD-fed mice. 

**Figure 5:**
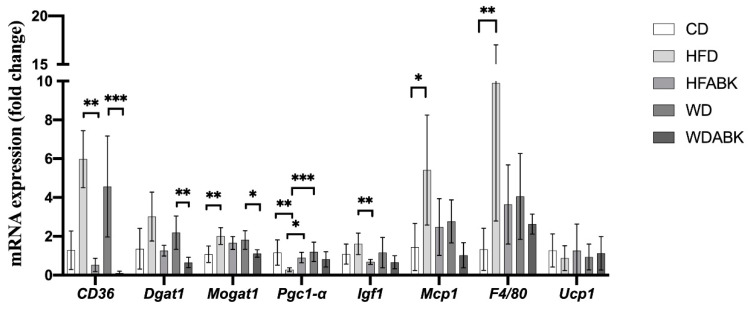
mRNA expression of adipogenesis- and inflammation-related genes in eWAT from HFD- and WD-fed mice. Data are expressed as mean ± SD. * *p* < 0.05, ** *p* < 0.01, *** *p* < 0.001. (n = 8 per group) Statistics were analyzed by using Kruskal–Wallis test with a post hoc Dunn’s multiple comparison test.

The eWAT has been shown to be particularly susceptible to inflammation in obese subjects [32]. Therefore, to understand the effect of AB-Kefir on the regulation of lipid metabolism, mRNA expression of adipogenesis- and inflammation-related genes in eWAT from test mice was determined using qPCR. As shown in Figure 5, administration of HFD significantly increased *Mogat1*, *Mcp1*, and *F4/80* and decreased *Pgc-1* mRNA expression compared to those in the CD group. In contrast, administration of AB-Kefir markedly reduced *CD36* and *Igf1* and increased *Pgc-1* mRNA expression. Additionally, the WD group did not show a significant change in these gene levels compared to those in the CD group. Notably, administration of AB-Kefir in WD-fed mice reduced the mRNA expression levels of *CD36*, *Dgat1*, and *Mogat1* compared to those in WD-fed mice not administered AB-Kefir. The *Ucp1* expression showed no significant difference between each group.

### 3.5. Administration of AB-Kefir Resulted in a Change in SCFA Production in WD-Fed Mice but Not in HFD-Fed Mice

**Figure 6:**
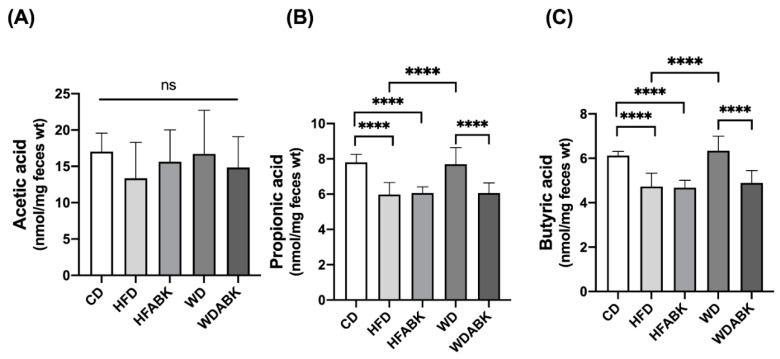
Effect of AB-Kefir on fecal SCFA level in HFD- and WD-fed mice. Level of (**A**) acetic, (**B**) propionic, and (**C**) butyric acid in feces are shown. Data are expressed as mean ± S.D. (n = 8 per group) **** *p* < 0.0001. Statistics were analyzed by using one-way ANOVA with Tukey’s post hoc test.

To evaluate the effect of AB-Kefir on changes in intestinal SCFA production, the major SCFAs, including acetic, propionic, and butyric acid, in the feces of test mice were measured using HPLC. As shown in Figure 6, HFD-fed mice exhibited a significant reduction in the levels of propionic acid and butyric acid compared with the CD group; however, administration of AB-Kefir did not alter the SCFA production in HFD-fed mice. In contrast, no change in SCFA production was observed in the WD group. Interestingly, administration of AB-Kefir to WD-fed mice markedly reduced the levels of propionic and butyric acid compared to those in the WD group not administered with AB-Kefir. 

### 3.6. AB-Kefir Impacted the Gut Microbiota Composition in HFD- and WD-Induced Obese Mice

**Figure 7:**
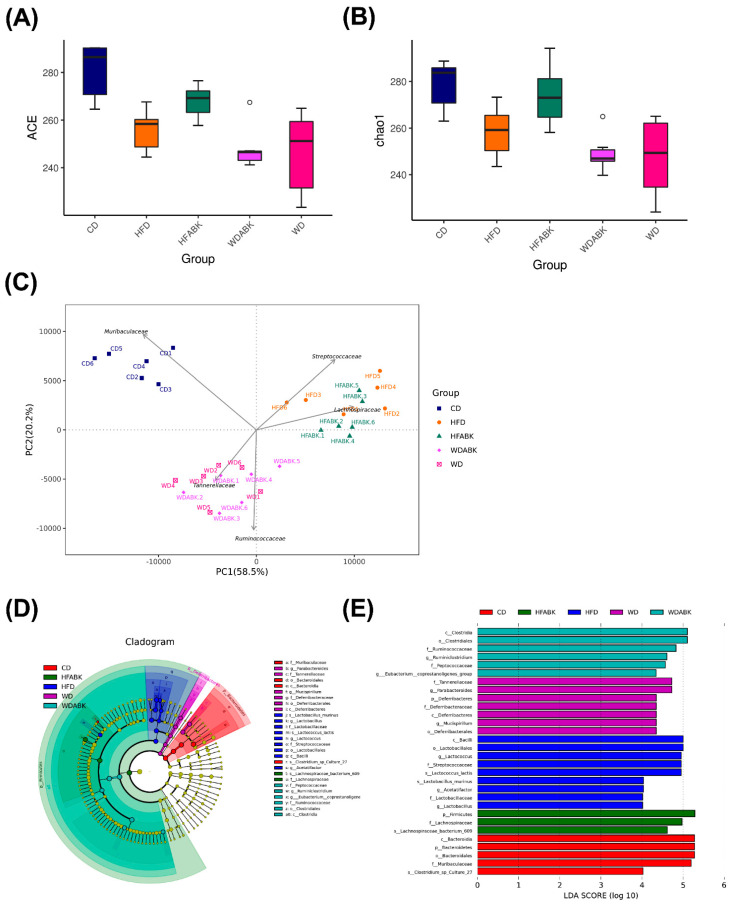
Effect of AB-Kefir on the gut microbiota composition in HFD- and WD-fed mice. (**A**) ACE, (**B**) Chao1 richness estimator, and (**C**) PCA plot of gut microbiota at the family level and the first two principal components, PC1 and PC2, are plotted. The five largest PCA loadings of taxa are indicated by arrows next to their genus names. (**D**,**E**) LEfSe comparison of the gut microbiota between CD, HFD, HFABK, WD, and WDABK groups. Each point represents one sample (n = 6 per group).

As AB-Kefir showed the ability to reduce the characteristics of obesity, we speculated whether AB-Kefir or AB-Kefir-modulated SCFAs made an impact on the gut microbiota composition. Stool samples were collected and analyzed using 16S rRNA gene sequencing. An average of 76,093 effective tags were obtained after qualify-filtering using the QIIME pipeline. To evaluate the diversity of microbiota composition, ACE and Chao1 indices were determined (Figure 7A,B). The ACE and Chao1 indices decreased in HFD and WD groups. The results indicated a downregulation in the richness of the gut microbiota richness in HFD- and WD-fed mice. Moreover, AB-Kefir upregulated the richness of microbiota in HFD-fed mice, but not in WD-fed mice. The reduction in the diversity of gut microbiota in obese mice was similar to the previous studies [33,34]. The weighted UniFrac PCA explained 58.5% and 20.2% of the variation in intestinal microbiota composition (Figure 7C). Although there was no significant difference in the gut microbiota α-diversity between the different diet treatments, the cluster of CD group was distinct from clusters of the HFD and WD groups. Moreover, clustering analysis at the family level of the gut microbiota indicated that the CD group was characterized by the presence of *Muribaculaceae*, HFD and HFABK by *Streptococcaceae* and *Lachnospiraceae*, and WD and WDABK by two other dominant families, namely *Tannerellaceae* and *Ruminococcaceae*. Obviously, the gut microbiota was more susceptible to diet than AB-Kefir treatment. Therefore, we further characterized high-dimensional biomarkers of gut microbiota among the mice in groups using LEfSe analysis with a 4.0-threshold value of LDA (Figure 7D,E). A taxonomy cladogram indicated that the phylum *Bacteroidetes* was abundant in the CD group; *Firmicutes* was abundant in HFD, HFABK, and WDABK groups; and *Deferribacteres* was abundant in WD group. *Muribaculaceae* was a potential microbiome biomarker found in the CD group. The species *Lactobacillus murinus* within the family *Lactobacillaceae* and *Lactococcus lactis* within the family *Streptococcaceae* were present in the HFD group. The species *Lachnospiraceae_bacterium_609* within the family *Lachnospiraceae* was markedly present in the HFABK group. The genera *Parabacteroides* within the *Tannerellaceae* family and *Mucispirillum* within the *Deferribacteraceae* family were significantly enriched in the WD group. Lastly, *Ruminiclostridium* within *Ruminococcaceae* and *Eubacterium_coprostanoligenes_*group were found in the WDABK group.

### 3.7. Correlation between Gut Microbiota and Characteristics of Obesity Affected by AB-Kefir Administration in HFD- and WD-Fed Mice

**Figure 8:**
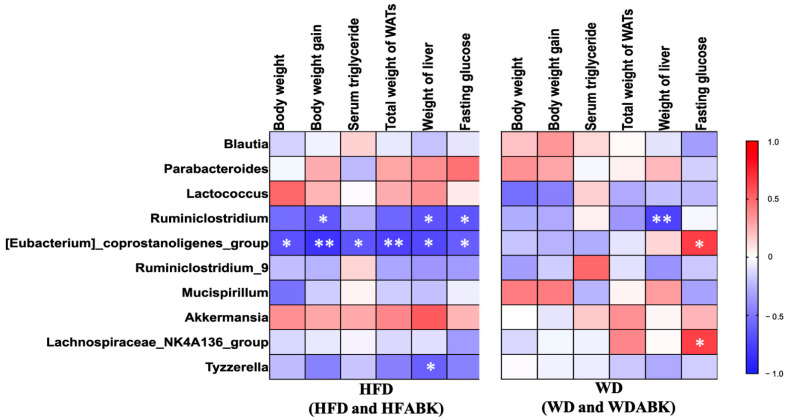
Spearman’s rank correlations between the top ten abundant bacterial genera and obesity-related parameters within HFD-fed mice (HFD and HFABK, n = 12) and WD-fed mice (WD and WDABK, n = 12). Spearman’s correlation analysis with an R value > 0.4 indicates a positive correlation (red color) and a value <−0.4 indicates negative correlation (blue color). * *p* < 0.05, ** *p* < 0.01.

To evaluate the correlation between gut microbiota and characteristics of obesity affected by AB-Kefir administration, the Spearman’s correlation coefficient between changes in six parameters (body weight, body weight gain, serum triglycerides, total weight of WAT, weight of liver, and fasting glucose) was obtained, and the top 10 most abundant intestinal genera in HFD- and WD-induced obese mice are shown in Figure 8. In HFD-fed mice (HFD and HFABK groups), the abundance of *Eubacterium_ coprostanoligenes_*group and *Ruminiclostridium* displayed a significant negative correlation with changes in body weight gain, liver weight, and fasting glucose. The abundance of *Tyzzerella* was negatively correlated with the weight of liver tissue. By contrast, in WD-fed mice (WD and WDABK groups), the change in fasting glucose was positively associated with *Eubacterium_coprostanoligenes_*group and *Lachnospiraceae_NK4A136_*group. In addition, the abundance of *Ruminiclostridium* was negatively correlated with the weight of liver tissue. 

## 4. Discussion

Diet has a major influence on the development of obesity and metabolic disorders. Consumption of different types of high-fat and high-sucrose foods leads to various metabolic disorders [35]. A spectrum of studies have shown that specific probiotic supplementation could effectively control obesity [7,36,37] and that multi-strain probiotics have better beneficial effects on prevention of the metabolic disorder [38]. However, the effect of multi-strain probiotics on different types of diet-induced characteristics of obesity and intestinal dysbiosis remains unclear. In this study, we demonstrated that administration of AB-Kefir showed a beneficial effect on reducing body weight gain, glucose intolerance, hepatic steatosis with a minor influence on gut microbiota composition in HFD-fed mice but not in WD-fed mice. In addition, AB-Kefir significantly reduced the weight and size of adipose tissue by regulating the expression of *CD36*, *Igf1*, and *Pgc1* in HFD-fed mice. Although AB-Kefir did not exhibit a positive effect on the volume of WAT, it significantly regulated *CD36*, *Dgat1*, and *Mogat1* mRNA expression in WD-fed mice.

High-fat and high-sucrose diets are often employed to develop obesity in animal models. The major factor contributing to the development of obesity is diet composition, rather than genetic background of mice [39]. In the present study, we observed that the changes in body weight and metabolic disorder in mice were more susceptible to HFD than WD. Similar results were observed in a number of studies that fed mice with excess fat or sucrose for a period of time [14,39,40]. Long term HFD caused an increase in the volume and size of the adipose tissue, whereas a high-sucrose diet induced severe glucose intolerance in both obese and lean mice [14]. Moreover, a high-lard diet induced a more severe metabolic disorder than a high-butter diet in mice [41]. Furthermore, a difference in the composition of the diet can affect the microbiota–host interactions; for instance, dietary cholesterol was associated with diet-induced obesity resistance in germ-free mice [42]. The fat in the WD and HFD used in this study was composed of butter and lard, respectively. Because butter has a higher amount of cholesterol than lard, the weight gain of the WD group was significantly lower than that of the HFD group, corroborating the results of a previous study, which suggests that dietary cholesterol protects germ-free mice from obesity [42]. Although the effect of lard and butter on host health is still controversial, our results showed that AB-Kefir intervention had more positive effects on the prevention of obesity in HFD-fed mice than in WD-fed mice, which implied that AB-Kefir might have aided in the reduction of diet-induced obesity-associated characteristics. However, the precise mechanism and effect of dietary composition on obesity need to be further studied. On the other hand, the type of fat in the diet may be an important factor to modulate SCFA levels in feces from test mice since butter in WD used in our study is the butyrate food source. Our observation was similar to a previous study showing that higher fecal SCFA levels after a high saturated fat diet was observed compared to baseline in human subjects [43]. The lower fecal propionate and butyrate level in the WDABK group may be due to higher absorption compared to the WD group. Future study should focus on the effect of AB-Kefir on different dietary fat absorption in the intestine thereby influencing the intestinal metabolism.

WAT is considered an endocrine organ that regulates appetite, immunity, lipid metabolism, and weight homeostasis in the host [44]. Several cellular markers, such as CD36 [45], Mogat1 [46], Igf1 [47], and Pgc1α [48], can influence adipocyte differentiation, adipogenesis, and triglyceride accumulation in WAT. CD36 activation increased free fatty acid uptake and inhibited leptin expression, resulting in triglyceride synthesis and fat storage in WAT [49]. Moreover, *Mogat1* and *Dgat1* expression activated by free fatty acids subsequently enhanced triglyceride accumulation [50]. In addition, Pgc1α and Igf1 expression regulated adipocyte differentiation, glucose homeostasis, and insulin sensitivity in WATs [48,51]. Thus, AB-Kefir regulated the genes related to fatty acid uptake (*CD36*), triglyceride synthesis (*Mogat1* and *Dgat1*), and glucose homeostasis (*Pgc1* and *Igf1*) in the WAT of mice. These results suggest that AB-Kefir effectively modulates multifaceted aspects of lipid metabolism in obese mice.

Functional associations between gut microbiota and health status of the host have been demonstrated previously [16,52,53]. Changes in gut microbiota result in modulation of host metabolism, such as energy expenditure [54] and production of SCFAs [52] and bile acids [53]. In our study, the administration of AB-Kefir exerted beneficial effects on obesity in HFD-fed mice. However, an absence of a significant difference in the cluster of gut microbiota in AB-Kefir groups compared to that in HFD and WD groups indicated that sample clustering was based on diet, although the AB-Kefir groups showed a higher diversity in the gut microbiota composition than that in the HFD group. Our results agreed with a previous study that diet may contribute towards the manipulation of the overall microbiome to a greater extent than probiotic intervention [55]. Therefore, we inferred that specific taxa in the gut microbiota may play a role in influencing the metabolic modulation. HFD-induced obesity characteristics were associated with an increased population of *L. lactis* and *L. murinus,* whereas AB-Kefir-induced improvement of obesity was associated with *Lachnospiracea*. *L. murinus* has been shown to have a positive correlation with markers of inflammation, such as IL-6 and MCP-1, in the fat of genetically different mice that were administered HFD [56]. *Lachnospiraceae* has been shown to produce beneficial metabolites, such as SCFAs, and maintain gut health [57,58], although SCFA production between HFD and HFABK groups showed no difference. We also evaluated Spearman’s correlations between bacterial genera and obesity-related parameters in HFD (combining HFD and HFABK) and WD groups (combining WD and WDABK), thereby allowing us to identify specific associations for each group. The results indicated that the relative abundance of *Eubacterium_coprostanoligenes_group* and *Ruminiclostridium* were negatively correlated with body weight gain, liver weight, and serum glucose levels in the HFD group. The genus *Eubacterium coprostanoligenes* was reported to decrease blood cholesterol concentration in germ-free mice [59] and increase gut microbiota content of obese mice supplemented with phosphatidylserine [60]. Diet-induced obesity resistance in mice fed cholesterol-rich diet was characterized by the presence of *Eubacterium coprostanoligenes* [42]. The metabolite coprostanol metabolized from cholesterol by *Eubacterium coprostanoligenes* had been shown to have a positive correlation with fecal bile acid level in a human trial [61,62]. Therefore, as *Eubacterium coprostanoligenes* plays an important role in controlling metabolic disorders, we presumed that this strain might have contributed to the effects of AB-Kefir in HFD-induced obesity in our study. The profile of bacterial metabolites in feces and serum of mice should be further determined in future study. The abundance of *Ruminiclostridium* is positively associated with fecal acetate in healthy adults [63]. By contrast, fasting serum glucose levels in mice with WD were positively correlated with *Eubacterium_coprostanoligenes_*group and *Lachnospiraceae_NA4K136_*group; however, the levels were negatively correlated with these two taxa in HFD-fed mice. In summary, AB-Kefir intervention in mice models of different diet-induced obesity characteristics showed specific manipulative effects on gut microbiota, which in turn were associated with attenuation of the metabolic syndrome. However, a number of limitations of this study should be addressed. The effect of AB-Kefir on the different gender needs to be evaluated, and the changes in the profile of metabolites, energy expenditure and intestinal fat absorption by AB-kefir in obesity treatment should be pursued in future research. Taken together, the findings of our study highlighted the specific effects of probiotics on diet-induced obesity and revealed that the use of probiotics in weight management was strongly influenced by diet. Therefore, in future, performing clinical trials to support these findings in humans is warranted.

## 5. Conclusions

This study demonstrated that AB-Kefir, containing six strains of lactic acid bacteria and a *Bifidobacterium* strain, showed beneficial effects by reducing body weight gain, glucose intolerance, and hepatic steatosis, as well as by influencing gut microbiota composition in HFD-fed mice, but not in WD-fed mice. In addition, AB-Kefir significantly reduced body fat in diet-induced obese mice by regulating the genes related to fatty acid uptake, triglyceride synthesis, and glucose homeostasis. Overall, this study provides critical evidence to understand the interactions between probiotics, gut microbiota, and diet in the treatment of obesity.

## Data Availability

The original contributions presented in the study are publicly available. This 16S rRNA data can be found at the NCBI under accession number PRJNA722813.

## Supplementary Materials

The following are available online at https://www.mdpi.com/article/10.3390/nu13072182/s1, Table S1. Formulation of diets. Table S2. The primer sequences used in this study.
