# Supplementary material for: AB-Kefir Reduced Body Weight and Ameliorated Inflammation in Adipose Tissue of Obese Mice Fed a High-Fat Diet, but Not a High-Sucrose Diet
Source: Nutrients. 2021 Jun 24;13(7):2182. doi: 10.3390/nu13072182 (PMC8308298; doi:10.3390/nu13072182)
Supplement: Supplementary file 1 [file nutrients-13-02182-s001.zip › nutrients-1235485-supplementary.pdf]

Table S1. Formulation of diets.

Diet	**D12450J**<br>10 kcal% CD	**D12492**<br>60 kcal% HFD	**D12079B**<br>Western Diet
**Caloric Information**			
Protein	20% kcal	20% kcal	17% kcal
Fat	10% kcal	60% kcal	40% kcal
Carbohydrate	70% kcal	20% kcal	43% kcal
Energy Density	3.82 kcal/g	5.21 kcal/g	4.67 kcal/g
**Ingredient**			
Casein	200.00 g	200.00 g	195.00 g
Methionine	0.00 g	0.00 g	3.00 g
L-Cystine,	3.00 g	3.00 g	0.00 g
Starch, Corn	506.20 g	0.00 g	0.00 g
Lodex 10	125.00 g	125.00 g	100.00 g
Sucrose	72.80 g	72.80 g	350.00 g
Fiber	50.00 g	50.00 g	50.00 g
Lard	20.00 g	245.00 g	0.00 g
Soybean Oil, USP	25.00 g	25.00 g	0.00 g
Butter, Anhydrous	0.00 g	0.00 g	200.00 g
Corn Oil	0.00 g	0.00 g	10.00 g
Mineral(S10001A)	50.00 g	50.00 g	17.50 g
Calcium Phosphate	0.00 g	0.00 g	17.50 g
Calcium Carbonate	0.00 g	0.00 g	4.00 g
Choline Bitartrate	2.00 g	2.00 g	2.00 g
Vitamin(V10001C)	1.00 g	1.00 g	1.00 g
Ethoxyquin	0.00 g	0.00 g	0.04 g
Cholesterol, NF	0.00 g	0.00 g	1.50 g
*Cholesterol from lard	0.02 g	0.23 g	0.00 g
#Cholesterol from butter	0.00 g	0.00 g	0.60 g

*Typical analysis of cholesterol in lard = 0.95 mg/gram

#Anhydrous milk fat typically contains approximately 0.3% cholesterol.

Table S2. The primer sequences used in this study

Gene	Forward Primer Sequence (5'->3')	Reverse Primer Sequence (5'->3')
Mouse Gadph	ACTCCACTCACGGCAAATTC	TCTCCATGGTGGTGAAGACA
Mouse CD36	ATTGGTCAAGCCAGCT	TGTAGGCTCATCCACTAC
Mouse Dgat1	TCCGTCCAGGGTGGTAGTG	TGAACAAAGAATCTTGCAGACGA
Mouse Mogat1	TTGTGCTTTGGGGTGCTATCA	CCACAGTGGGAACTCTCCA
Mouse Pgc1α	CGATGTGTCGCCTTCTTGCT	CGAGAGCGCATCCTTTGG
Mouse Igf1	GCTTGCTCACCTTTACCAGC	AAATGTACTTCCTTCTGGGTCT
Mouse Mcp1	AGGTCCCTGTCATGCTTCTG	TCATTGGGATCATCTTGCTG
Mouse F4/80	TTTCCTCGCCTGCTTCTTC	CCCCGTCTCTGTATTCAACC
Mouse Ucp1	ACTGCCACACCTCCAGTCATT	CTTTGCCTCACTCAGGATTGG

Gadph: Glyceraldehyde-3-phosphate dehydrogenase; **Dgat1**: Diacylglycerol O-acyltransferase 1; **Mogat1**: Monoacylglycerol O-Acyltransferase 1; **Pgc1α**: Peroxisome proliferator-activated receptor gamma coactivator 1α; **Igf1**: Insulin-like growth factor 1; **Mcp1**: Monocyte chemoattractant protein 1; **Ucp1**: uncoupling protein 1.
